# The effects of increased dietary protein yogurt snack in the afternoon on appetite control and eating initiation in healthy women

**DOI:** 10.1186/1475-2891-12-71

**Published:** 2013-06-06

**Authors:** Laura C Ortinau, Julie M Culp, Heather A Hoertel, Steve M Douglas, Heather J Leidy

**Affiliations:** 1 Department of Nutrition & Exercise Physiology, University of Missouri, 204 Gwynn Hall, Columbia, MO 65211, USA; 2 General Mills Bell Institute of Health and Nutrition, Minneapolis, MN, USA; 3 School of Medicine, University of Missouri, 204 Gwynn Hall, Columbia, MO 65211, USA

**Keywords:** Snacking, High protein, Voluntary meal request, Satiety, Eating initiation

## Abstract

**Background:**

A large portion of daily intake comes from snacking. One of the increasingly common, healthier snacks includes Greek-style yogurt, which is typically higher in protein than regular yogurt. This study evaluated whether a 160 kcal higher-protein (HP) Greek-style yogurt snack improves appetite control, satiety, and delays subsequent eating compared to an isocaloric normal protein (NP) regular yogurt in healthy women. This study also identified the factors that predict the onset of eating.

**Findings:**

Thirty-two healthy women (age: 27 ± 2y; BMI: 23.0 ± 0.4 kg/m^2^) completed the acute, randomized crossover-design study. On separate days, participants came to our facility to consume a standardized lunch followed by the consumption of the NP (5.0 g protein) or HP (14.0 g protein) yogurt at 3 h post-lunch. Perceived hunger and fullness were assessed throughout the afternoon until dinner was voluntarily requested; ad libitum dinner was then provided. Snacking led to reductions in hunger and increases in fullness. No differences in post-snack perceived hunger or fullness were observed between the NP and HP yogurt snacks. Dinner was voluntarily requested at approximately 2:40 ± 0:05 h post-snack with no differences between the HP vs. NP yogurts. Ad libitum dinner intake was not different between the snacks (NP: 686 ± 33 kcal vs. HP: 709 ± 34 kcal; p = 0.324). In identifying key factors that predict eating initiation, perceived hunger, fullness, and habitual dinner time accounted for 30% of the variability of time to dinner request (r = 0.55; p < 0.001).

**Conclusions:**

The additional 9 g of protein contained in the high protein Greek yogurt was insufficient to elicit protein-related improvements in markers of energy intake regulation.

## Background

Over the past 30 years, there has been a significant increase in the number of eating occasions beyond the standard ‘three meals a day’ [1-3]. In fact, approximately 30% of daily intake is comprised of snacking, which is defined as any eating occasion outside of a typical meal time [1,3-5]. Additionally, women tend to snack more frequently than men [6]. Over the past 30y, yogurt has become a popular afternoon snack [7]. More recently, Greek yogurt was introduced into the U.S. market which has led to further increases in yogurt consumption [8]. One of the unique qualities of Greek yogurt is the higher quantity of dietary protein compared to regular yogurt (20–25 vs. 5-7 g protein/serving, respectively) [9].

Increased dietary protein has been shown to be an effective dietary strategy to promote improvements in appetite control, satiety, and the regulation of energy intake [10-13]. However, the majority of existing data comes from the comparison of normal protein vs. high protein meals consisting of 240-1400 kcal/meal, containing 35-50 g protein [11,12,14,15]. Of the high protein snack studies published to date, many include foods containing large quantities of protein (i.e., 20-46 g) [16-19]. Further, these foods were either not typically consumed as snacks (e.g. chicken breast) or not commercially available (e.g. whey-enriched water) [16,17,19].

The purpose of this study was to evaluate the impact of a higher-protein afternoon snack on appetite control, delays in eating initiation, and subsequent energy intake compared to an isocaloric normal protein snack in healthy women. This study also identified key factors that predict the onset of eating.

## Methods

### Participants

Thirty-two women were recruited through the University of Missouri list-serve. Participants were healthy, non-smoking women (age: 27 ± 2y; BMI: 23 ± 0.4 kg/m^2^) who habitually consumed yogurt as an afternoon snack (3 ± 1 occasions/wk). A medical history questionnaire was used as a screening tool to identify known food allergies, clinically-diagnosed eating disorders, diabetes, and rapid weight loss/gain (≥ 10 lbs) in the past six months. All participants were informed of the study procedures and potential risks. Written consent was obtained from all participants. The study procedures were approved by the University of Missouri’s (MU) Human Subjects Institutional Review Board. Participants received $150 for completing all study procedures.

### Experimental design

The study incorporated an acute, randomized, crossover-design comparing afternoon yogurt snacks containing either normal protein (5 g, NP) or higher-protein (14 g, HP) (Table 1). The participants were acclimated to each snack pattern for 3 consecutive days at home/work. On day 4 of each pattern, the participants consumed a standardized 300 kcal breakfast (15% protein/60% CHO/25% fat), at home, and reported to the Brain Imaging Center 1 h prior to lunch to begin the 8 h testing day. Each participant was placed in a separate room, absent of time cues. During the testing days, the participants were permitted to relax and engage in numerous (sedentary) activities. Some of these include reading, watching movies, homework etc. as long as the task was devoid of time cues. The testing day began with the consumption of the standardized 500 kcal lunch meal (15% protein/55% CHO/30% fat) and 8 oz water. The respective snack pattern was completed 3 h after lunch and the participants had 15 min to consume the snack and 8 oz water. Snack palatability and sensory characteristics (i.e., appearance, aroma, flavor texture and overall liking) were assessed for each snack using visual analogue scales (VAS). The questions were worded as “how strong is the… or how much do you like the…” with anchors at extremely dislike/low or extremely like/high. Validated perceived appetite (hunger, desire to eat, prospective food consumption, & fullness) questionnaires incorporating a 100 mm horizontal line rating scale for each response were given randomly as well as incrementally (every 30 min) using computerized VAS throughout the afternoon [20-22]. Only the 30 min time intervals were entered and reported.

**Table 1:** Snack characteristic

	**Normal**	**Higher**
	**Protein**	**Protein**
	**(NP)**	**(HP)**
Yogurt Characteristic	Regular	Greek-style
Serving Size (g)	170	170
Energy Content (kcal)	160	160
Energy Density (kcal/g)	0.94	0.94
Total Protein (g)	5.0	14.0
Total CHO (g)	30.0	25.0
Total Fat (g)	1.5	0
Snack Palatability (mm)		
Overall Liking	75 ± 3	72 ± 4
Appearance	75 ± 3	69 ± 3
Aroma	74 ± 3	66 ± 3*
Flavor	78 ± 3	70 ± 4
Texture	67 ± 4	68 ± 4

* Paired sample *t*-test; P < 0.05.

### Time to diner request & ad libitum dinner

At the beginning of each testing day, the participants were informed that they would be periodically asked whether they were ready to eat again (after the snack). However, they were also permitted to request to eat any time in between these questions. When the response was “yes, I want to eat dinner right now”, the time from snack consumption was recorded. The time to dinner approach has been utilized in several studies and is an excellent measure of the “satiety power” of meals/snacks [16,23-25]. Upon “voluntary dinner request,” the participants were presented with an ad libitum dinner of chicken parmesan pizza pockets cut into bite size pieces. Regardless of time of dinner request, the participants were required to remain in the facility until the full 8 h testing day was completed.

### Data and statistical analysis

Power calculations from previous snack studies [16,17,19,23,24] indicated that a sample size of n = 32 would yield 80% power to detected differences in perceived hunger, fullness, and time of dinner request between snack patterns. For perceived hunger and fullness measures, a repeated measure ANOVA was performed to determine main effects of time, snack condition, and interactions. Additionally, post-snack Area Under the Curve (AUC) for perceived hunger and fullness were calculated using the trapezoidal rule [26]. Paired-sample t-tests were applied to compare differences in snack palatability and sensory characteristics, post-snack AUC for perceived sensations, time to dinner request, and dinner intake between snack conditions. To examine the predictors of eating initiation, Pearson correlation analyses were performed on each post-snack time point for the following continuous variables: perceived hunger, fullness, desire to eat, and actual time of day as well as the categorical variable, habitual dinner time, which was collected from screening food records. For the later variable, at each time point, we enter ‘Yes’ this is the habitual time in which the participant typically eats dinner or ‘No’ this is not the habitual time in which the participant typically eats dinner. These variables were correlated with another categorical variable, time to dinner request. For this variable, at each time point, we entered either ‘Yes’ dinner was requested or ‘No’ dinner was not requested. A multivariate regression was then performed using the previously stated variables found to be associated with time to dinner request. All analyses were conducted using the Statistical Package for the Social Sciences (SPSS; ver.19; Chicago, IL). Significance was set as p < 0.05. All data is reported as mean ± SEM.

## Results

**Figure 1:**
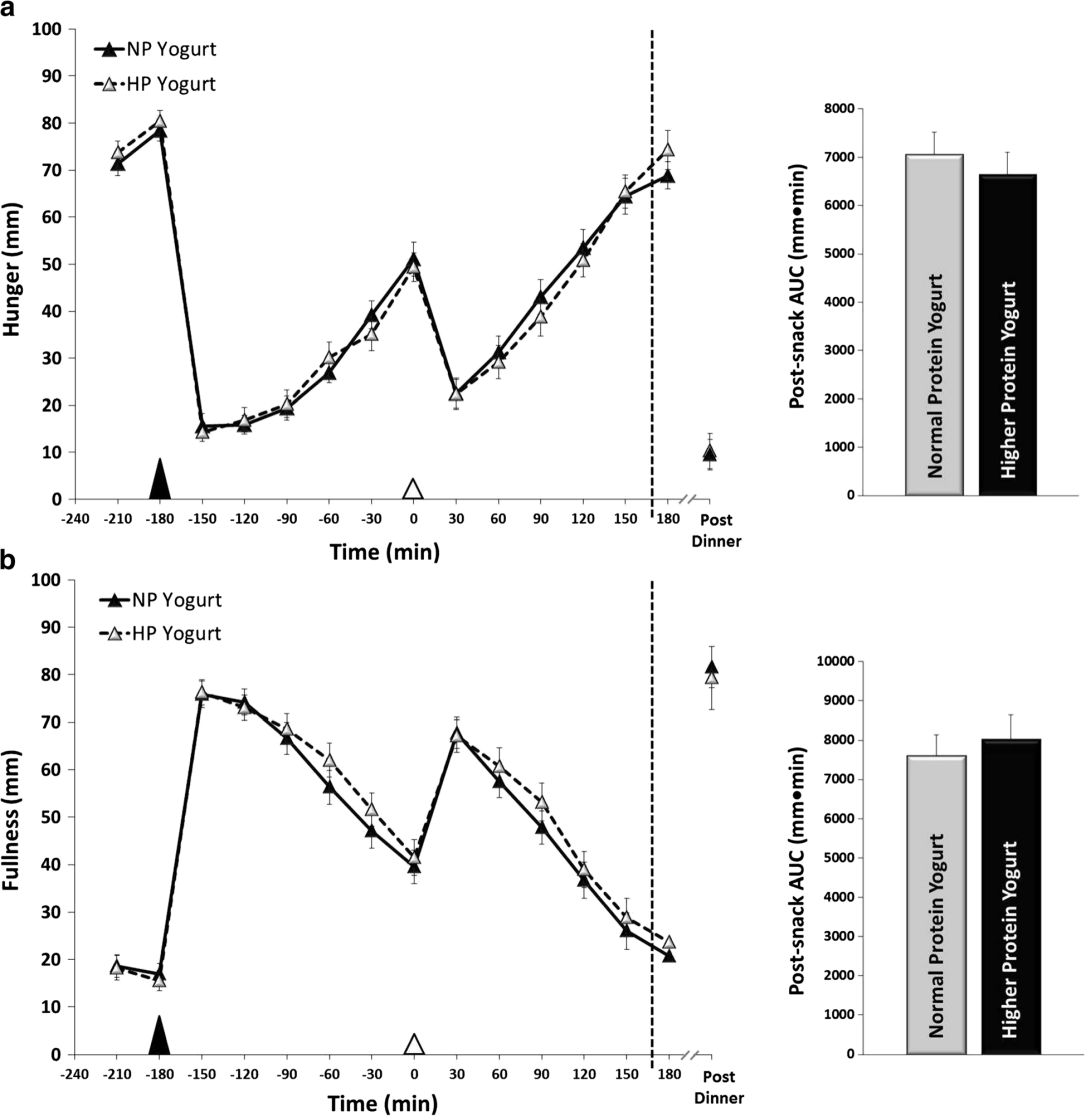
**Appetite and satiety.** Perceived (**a**) hunger and (**b**) fullness assessed throughout the 8 h testing day in 32 healthy women. The time of voluntary dinner request was variable between participants; thus, the data points on the graph, particularly after 2 h post snack, do not contain all 32 volunteer but only those who had not requested dinner yet. Voluntary dinner request is indicated on the graphs as a vertical dotted line; The average time to dinner request for Normal Protein (NP) and Higher Protein (HP) yogurt snacks was 2:43 ± 0:06 and 2:41 ± 0.04 h, respectfully. The post-snack area under the curve (AUC) is shown to the right of the line graphs. The ▲ represents the standardized lunch meal, whereas the Δ represents the NP and HP afternoon yogurt snacks.

Figures 1a&b illustrate the perceived hunger and fullness responses throughout the testing days until dinner was voluntarily requested. Snacking led to an immediate reduction in perceived hunger which was sustained 1:30 h post-snack (time effect, p < 0.001.) Similar responses were also observed with desire to eat and prospective food consumption (data not shown). Snacking also led to immediate increases in perceived fullness which was sustained 1:30 h post-snack (time effect, p < 0.001). No differences in post-snack hunger or fullness AUC were observed between the NP and HP yogurt snacks (Figures 1a&b). No differences in post-snack desire to eat or prospective food consumption AUC were observed between snacks (data not shown). No differences were detected in eating initiation (i.e., time to dinner request) between the NP (2:43 ± 0:06 h) vs. HP (2:41 ± 0:04 h; p = 0.757). No differences in ad libitum dinner intake were found between snacks (NP: 686 ± 33 kcal vs. HP: 709 ± 34 kcal; p = 0.324).

In examining the potential factors that contribute to the onset of eating, a multivariate regression analyses revealed that hunger, fullness, and habitual dinner time were all predictors of time to dinner request, accounting for 30% of the variability (r = 0.55; p < 0.001). Further, 95% of the participants requested dinner when perceived hunger was between 74-82 mm on the VAS questionnaire.

## Discussion

No differences in afternoon appetite control, eating initiation, or subsequent food intake were observed when comparing two commonly consumed, relatively small (160 kcal) afternoon yogurt snacks, varying in protein content (5 g vs. 14 g protein). In a previous snack study comparing low protein (~5 g protein) vs. high protein (~20 g protein) snacks of 20–80 kcal also found no differences in post-snack hunger, desire to eat, prospective food consumption, or fullness [19]. The lack of differences observed in this study as well as the current study might be due to either the small energy content of the snacks consumed or the relatively small difference in protein content between the low vs. high protein snacks (5-15 g protein differential between snacks) [19]. In another study that incorporated a larger snack (i.e. 287 kcal) with similar protein quantities (3 vs. 10 g) as the previously discussed and current study, consumption of the snack with 10 g protein resulted in decreased hunger and increased fullness up to 60 min post-snack. This study also observed a lack of difference in time to dinner request between the two snacks [27]. An additional study that incorporated larger snacks (i.e. 240 kcal) with greater protein quantities (26–46 g protein), substantial increases in hunger and reductions in fullness were observed compared to normal protein versions [16]. Because a larger snack size (i.e. energy content) typically occurs simultaneously with larger protein quantities, it is unclear as to which factor has the greatest impact on appetite control and satiety.

In addition to perceived sensations of fullness over time, another indicator of satiety includes the onset of subsequent eating (i.e., eating initiation). Most of the current research utilizes a fixed meal design resulting in the consumption of a subsequent meal without taking into consideration the motivational state of the individual (i.e., whether the individual is sufficiently hungry enough to want to eat again and/or would have requested dinner at that time) [17-19]. Only a limited number of studies, including the current study, incorporate this approach by identifying the time to voluntary dinner request [16,25,27,28]. Of these, only a subset were snack studies [16,27], and only one study previously evaluated the effects of normal vs. high protein snacks [16]. As shown in Marmonier, et al. [16], a high protein afternoon snack led to a 30 min delay in voluntary request for dinner compared to consuming high carbohydrate or high fat snacks [16]. The lack of difference in the onset of subsequent eating in the current study might again be attributed to the smaller absolute protein quantities (5 vs. 14 g) or the smaller protein differential (9 g) between our snacks compared to the Marmonier et al. study which compared 26 g vs. 46 g protein with a 20 g differential. Therefore, dose-dependent studies of varying protein quantities in varying snack sizes are warranted.

Lastly, by allowing the timing of dinner to fluctuate, we were able to determine that perceived hunger is the best predictor of voluntary eating initiation. Further, in healthy women, perceived hunger reaching a value of approximately 80 mm was sufficient to elicit the onset of eating. Thus, it is now possible to incorporate this data into fixed meal designs based on a specific hunger threshold.

### Limitations

In the current study, several factors exist which might have influenced the overall study findings. Dietary restraint was not specifically assessed, or used as part of the screening criteria. However, individuals who were clinically diagnosed with an eating disorder, those that displayed rapid weight gain and/or loss over a short period of time (≤ 6 months), those that had a-typical eating behaviors and/or patterns (1 meal/day; 6 meals/day, etc.), or those who infrequently or never snacked were excluded from the study. We sought to include a sample size that would be representative of a healthy female population even though it might have included restrained eaters. Menstrual cycle phase was also not controlled. Currently there is conflicting and limited data as to the extent that menstrual cycle phase influences acute appetite control and food intake [29-32]. Recent data from our laboratory suggests that there is no effect of menstrual cycle on these outcomes [33]. However, by randomizing the order of the snack conditions between subjects, it is likely that we had an equal distribution of testing days that fell in the follicular and/or luteal menstrual phases. The relatively small energy protein differential of these snacks may not be sufficient enough to elicit protein-related improvements in appetite control, delays in eating initiation, and subsequent energy intake. Lastly, we recognize that these results are specific to healthy premenopausal women and cannot be extrapolated to other populations. Thus, further research to determine a “protein threshold” to elicit the proposed protein-related improvements in a more diverse population is warranted.

## Conclusions

No differences in post-snack appetite control, satiety, eating initiation, or subsequent food intake were found when comparing two commonly consumed afternoon yogurt snacks, varying in protein content. Thus, these data suggest that the additional 9 g of protein contained in the higher protein Greek yogurt was insufficient to elicit protein-related improvements in appetite control and satiety in healthy women.

## Abbreviations

NP: Normal Protein; HP: Higher-Protein; AUC: Area Under the Curve.

## Competing interests

HJL, LCO, HAH, and SMD have no conflict of interest. JMC is employed by the General Mills Bell Institute of Health and Nutrition.

## Authors’ contributions

HJL and JMC designed the research; HJL, LCO, HAH, and SMD conducted the research; HJL and LCO analyzed the data; HJL and LCO wrote the paper; and HJL had primary responsibility for the final content. All authors read and approved the final manuscript.
